# High-grade tumor budding is a risk factor for survival in patients with laryngeal squamous cell carcinoma

**DOI:** 10.1016/j.bjorl.2023.101310

**Published:** 2023-08-22

**Authors:** Li Luo, Honggang Liu

**Affiliations:** a Capital Medical University, Beijing Tongren Hospital, Beijing Key Laboratory of Head and Neck Molecular Diagnostic Pathology, Department of Pathology, Beijing, China; b Capital Medical University, Beijing Luhe Hospital, Department of Pathology, Beijing, China

**Keywords:** Laryngeal squamous cell carcinoma, Tumor budding, T1-2N0, Prognosis

## Abstract

**Objective:**

With the increasing incidence and mortality of laryngeal squamous cell carcinoma worldwide, researchers continue to search for novel prognostic factors and treatment methods for preventing early laryngeal squamous cell carcinoma from becoming advanced laryngeal squamous cell carcinoma. This study aims to determine if tumor budding is an independent risk factor associated with the survival of patients with laryngeal squamous cell carcinoma.

**Methods:**

268 cases of laryngeal squamous cell carcinoma were studied, and tumor budding was analyzed for associations with clinicopathological features and clinical outcomes.

**Results:**

Tumor budding was divided into low-grade tumor budding (0–6/0.785 mm^2^) and high-grade tumor budding (≥7/0.785 mm^2^) based on the results of the receiver operating characteristics curve analysis. Logistic regression analysis showed that smaller tumor cell nests, the low levels of tumor-infiltrating lymphocytes, and higher pathological T staging were the risk factors for high-grade tumor budding (*p* < 0.05). In the low-grade tumor budding group, there was no statistic difference in survival between patients without tumor budding and those with 1–6/0.785 mm^2^ tumor budding. Multivariate survival analysis showed high-grade tumor budding (*p* < 0.001) was independent prognostic factors for disease-free survival and overall survival in laryngeal squamous cell carcinoma. High-grade tumor budding was also an independent prognostic factor for disease-free survival (*p* = 0.037) and overall survival (*p* = 0.009) in T1-2N0 laryngeal squamous cell carcinoma.

**Conclusions:**

Smaller tumor cell nests, the low levels of tumor-infiltrating lymphocytes, and higher pathological T staging were closely associated with high-grade tumor budding in laryngeal squamous cell carcinoma. High-grade tumor budding may be an adverse risk factor that affects not only the disease-free survival and overall survival of laryngeal squamous cell carcinoma patients but also the survival of T1-2N0 laryngeal squamous cell carcinoma patients.

**Level of Evidence:**

Level 4.

## Highlights

• Novel prognostic factors need to be developed in laryngeal cancer.
• Tumor budding is related to smaller tumor cell nest in laryngeal cancer.
• Tumor budding is related to higher pT stage in laryngeal cancer.
• Tumor budding is related to the low levels of TILs in laryngeal cancer.
• Tumor budding may predict recurrence and death of laryngeal cancer patients.

## Introduction

Laryngeal Squamous Cell Carcinoma (LSCC) is one of the most common malignant tumors of the upper aerodigestive tract. From 1990 to 2017, the numbers of cases and deaths of LSCC increased by 58.7% and 33.9%, respectively, worldwide,^1^ and the 5 year overall survival rate of LSCC patients was about 64.0%.^2^ Even with continual advancements in the diagnosis and treatment methods of LSCC, however, the 2 year or 5 year observed survival or relative survival of LSCC patients have not been significantly improved due to a large recent increase in the proportion of stage 4 LSCC patients. Hence, there is an urgent need to identify the patients with poor prognoses, and treat early LSCC actively in order to prevent early LSCC from worsening to advanced LSCC.^3^ Although some new prognostic indicators such as nodal extracapsular extension and infiltrating growth patterns^4^ can help to identify LSCC patients facing poor prognoses, the existing prognostic evaluation system still needs to be constantly replenished and updated for predicting the survival of LSCC and guiding treatment.

Tumor Budding (TB) occurs when there is a small tumor cell cluster with between 1 and 5 tumor cells distributed at the invasive front of the tumor. In 1985, Gabbert^5^ found some isolated tumor cells at the invasion front of differentiated and undifferentiated colon carcinoma, which are all closely related to the dedifferentiation process of tumor cells, and enable tumor cells to break away from the tumor and have the ability to migrate. Later studies have confirmed that TB may mimic the process of tumor cell epithelial mesenchymal transformation^6^ and that it is closely related to angiolymphatic invasion, lymph node metastasis, and shorter survival. Thus, TB has become an independent prognostic risk factor for a variety of solid tumors, including colorectal cancer,^7^ gastric cancer,^8^ lung cancer,^9^ esophageal cancer,^10^ and breast cancer.^11^ However, studies of TB in head and neck squamous cell carcinoma mainly focus on the oral cavity and tongue,^6^, ^12^, ^13^ and there are few studies on LSCC and few enrolled cases. The relationship between TB and the clinicopathological parameters and survival of LSCC are still unclear, and there are no studies of TB in T1-2N0 early LSCC. Our study therefore aims to investigate the relationship between TB and the clinicopathological parameters and prognoses of patients with LSCC and T1-2N0 LSCC.

## Methods

### Study design and case selection

The retrospective study included 366 patients diagnosed with primary LSCC who underwent radical surgical resection of LSCC and cervical lymph node dissection from 2004 to 2021 in Beijing Tongren Hospital and Beijing Luhe Hospital. The exclusion criteria included patients with a history of other malignancies; patients with a histologic type other than squamous cell carcinoma; patients who had received neoadjuvant therapy before surgery; and patients with incomplete clinical information and follow-up data. According to the inclusion and exclusion criteria, 268 patients were included in our study cohort. Informed consent was obtained from patients. The approval of our study was granted by the Ethics Committee. Our study has been carried out in accordance with the Code of Ethics of the Word Medical Association (Declaration of Helsinki) for experiments involving humans.

### Histologic assessment

All Hematoxylin and Eosin (HE) stained sections of the 268 LSCC patients and their histological features were reevaluated under a Nikon microscope by two experienced head and neck pathologists who were blinded to the patients’ clinicopathological information, including tumor histologic grade, tumor cell nest size, tumor cell mitotic activity, tumor stroma ratio, stromal tumor Infiltrating Lymphocytes (TILs), TB activity, pathological-T (pT) stage, pathological-N (pN) stage, angiolymphatic invasion, and perineural invasion. Any differences of opinion between the two pathologists were discussed under a multi-head microscope until a consensus was reached.

Two sections were taken from one tumor tissue block at 50 microns interval, and about 10 sections were used for histologic evaluation in each laryngeal cancer case. The modified version of Broders’ grading system used for evaluating the histologic grade. 0%‒25% of undifferentiated tumor cells and 75%–100% of differentiated tumor cells were well-differentiated tumors; 50 %–75 % of differentiated tumor cells and 25%‒50% of undifferentiated tumor cells were moderately differentiated tumors; more than 50% of undifferentiated tumor cells were poorly differentiated tumors. The overall tumor cell nest size was evaluated in tumor areas containing the smallest tumor cell nests based on Boxberg's methods,^14^ rather than in the front of the tumor invasion, to avoid confusion with TB. The tumor cell nests consisting of less than 5 tumor cells were called small cell nests, 5–15 tumor cells were called intermediate cell nests, and more than 15 tumor cells were called large cell nests. High mitotic count was defined as greater than 15 mitotic counts in 10 consecutive High Power Fields (HPFs), and low mitotic count was defined as less than or equal to 15 in 10 HPFs.^15^ The tumor stroma ratio was divided into two groups using a cutoff of less than 50% for the low group and greater than or equal to 50% for the high group.^16^ In addition, the stromal TILs were divided into 0%‒10% TILs, 11%‒50% TILs, and greater than 50% TILs.^17^

TB was evaluated based on the Colorectal Cancer Tumor Budding Report recommendations of the International Tumor Budding Consensus Conference (ITBCC) 2016^18^ and was assessed using a single 10× objective microscope at the hot spot areas showing the maximal budding at the front of the tumor invasion. Then, a 20× objective was used with a 22 mm field diameter, and the results were divided by the normalization factor (1.210 based on a 22 mm eyepiece field diameter) to obtain the standardized budding count. Finally, pathological staging of LSCC was performed according to the eighth edition of the American Joint Committee on Cancer (AJCC) staging system.

### Outcomes

The 268 LSCC patients were followed up for 10–192 months, and the end outcomes were Overall Survival (OS) and Disease-Free Survival (DFS). DFS was assigned as the period of clinical, radiological, or pathological metastasis or recurrence after surgery or the period until the last follow-up, and OS was defined as the time from surgery to the date of death or final follow-up.

### Statistical analysis

All statistical analysis was performed using Statistics Package for Social Sciences (SPSS) software, version 21.0 (IBM Corporation, NY, USA). In addition, a Receiver Operating Characteristics (ROC) curve analysis was performed to determine the cutoff point of TB grouping. Associations between clinicopathological parameters and TB were assessed with Chi-Square tests, and Logistic regression analysis was used to analyze the clinicopathological risk factors related to TB. The survival curves were plotted using the Kaplan-Meier method, and the Log-rank test assessed the survival difference. Univariate and Multivariate survival analysis were also performed using a Cox proportional hazard model; *p*-values lower than 0.05 were considered to indicate statistically significant results for all statistical tests.

## Results

### General characteristics

The ages of the 268 patients ranged from 35 to 86 years old, with a median of 62 and an average age of 61.96 ± 9.117. According to the median age, 142 patients (53.0%) were less than 62 years old, and 126 patients (47.0%) 62 years old or older. There were 240 males (89.6%) and 28 females (10.4%). Additionally, well-differentiated tumors were present in 64 of the cases (23.9%), moderately differentiated tumors in 142 cases (53.0%), and poorly differentiated tumors in 62 cases (23.1%). Small tumor cell nests were observed in 47/268 cases (17.5%), intermediate cell nests in 162/268 (60.5%), and large cell nests in 59/268 (22.0%). Next, high mitotic figures occurred in 106/268 cases (39.6%) and low mitotic figures in 162/268 cases (60.4%). There were 153 cases (57.1%) with tumor stroma ratios ≤50% and 115 (42.9%) with tumor stroma ratios >50%. 77 cases (28.7%) were pT1 stage, 82 (30.6%) were pT2 stage, 70 (26.1%) were pT3 stage, and 39 (14.6%) were pT4 stage. Furthermore, 214 cases (79.9%) were pN0 stage, and 54 cases (20.1%) were pN1+N2 stage. There was angiolymphatic invasion in 49 cases (18.3%) and perineural invasion in 23 cases (8.6%). Finally, stromal TILs were divided into TILs 0%‒10% (80/268, 29.9%), TILs 11%‒50% (137/268, 51.1%), and TILs >50% (51/268, 19.0%).

**Figure 1:**
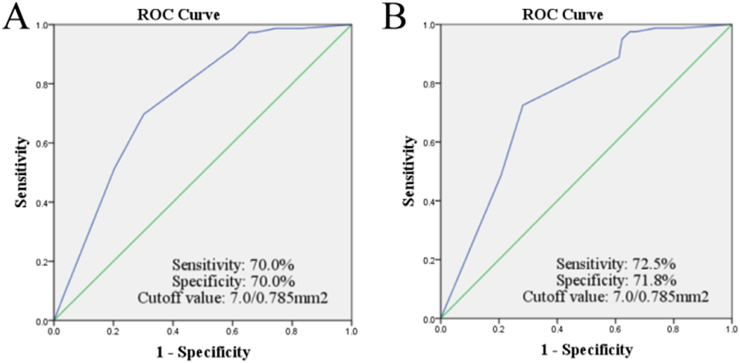
Tumor budding grouping by Receiver operating characteristics curve. Receiver operating characteristics curve showed the best cutoff point for association of numbers of tumors budding with recurrence (A) and death (B). The best cutoff point was seven buddings in 0.785 square millimeters.

**Figure 2:**
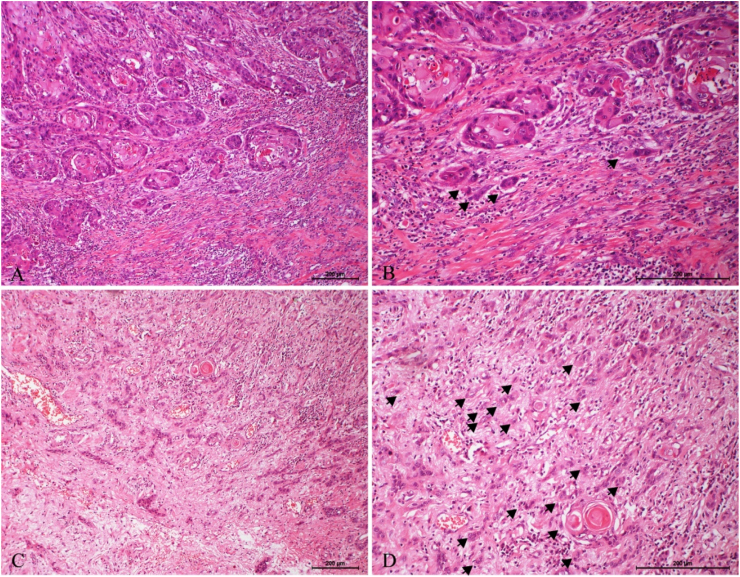
Examples of LSCC cases with low- and high-grade tumor budding. (A) An example with low-grade tumor budding (<7 buddings), where an invasive front arranged in large nests of cells with occasional presence of tumor budding is observed (Hematoxylin and eosin staining, ×100). (B) Low-grade budding (black arrow) under 200× magnification (Hematoxylin and Eosin staining). (C) Example with high-grade tumor budding (≥7 buddings), where the invasive front of the tumor is observed on the left where in the area of invasion there are abundant individual cells infiltrating the stroma (Hematoxylin and Eosin staining, ×100). (D) High-grade budding (black arrow) under 200× magnification (Hematoxylin and Eosin staining).

TB was observed in 226/268 (84.3%) cases, and the TB activity was 0–19/0.785 mm^2^, with an average of 6.46 ± 3.866/0.785 mm^2^ and a median of 7/0.785 mm^2^. ROC curve analysis showed that 7/0.785 mm^2^ was the optimal cutoff point to predict both the recurrence (sensitivity = 70.0%, specificity = 70.0%) and death (sensitivity = 72.5%, specificity = 71.8%) of the LSCC patients (Fig. 1). Thus, we divided the TB results into low-grade TB (0–6/0.785 mm^2^) (Fig. 2A and B) and high-grade TB (≥7/0.785 mm^2^) (Fig. 2C and D). Of the 268 cases, low-grade TB accounted for 81 (30.2%), and high-grade TB accounted for 187 (69.8%). In the low-grade TB group, there were 42 cases without TB, and 39 cases with 1 to 6 TB.

### Correlation between tumor budding grade and clinicopathological parameters

**Figure 3:**
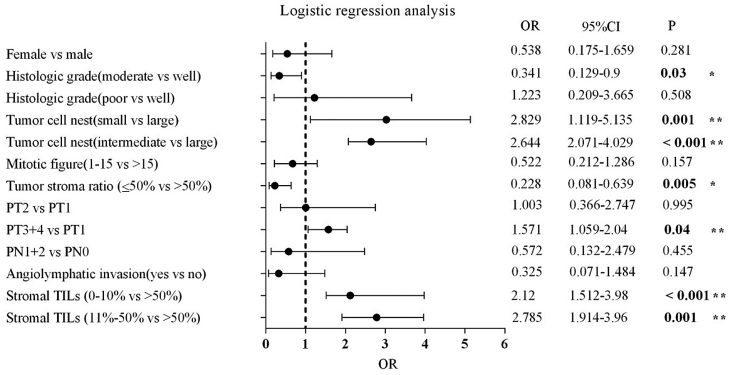
Logistic regression forest plot. Logistic regression forest plot showed small and intermediated tumor cell nest, higher pT stage, stromal TILs 0%‒10% and 11%‒50% were the risk factors (**) for high-grade budding. Moderately differentiated tumor and tumor stromal ratio ≤50% were the protective factors (*) for high-grade budding.

High-grade TB was associated with gender, histologic grade, tumor cell nest size, mitotic count, tumor stroma ratio, pT stage, pN stage, stromal TILs, and angiolymphatic invasion (*p* <  0.05). Logistic regression analysis further showed that intermediate (OR = 2.644, 95% CI 2.071–4.029, *p* < 0.001) and small tumor cell nest (OR = 2.829, 95% CI 1.119–5.135, *p* =  0.001), higher pT stage (OR = 1.571, 95% CI 1.059–2.040, *p* =  0.040), stromal TILs 0%‒10% (OR = 2.120, 95% CI 1.512–3.980, *p* <  0.001) and stromal TILs 11%‒50% (OR = 2.785, 95% CI 1.914–3.960, *p* = 0.001) were the risk factors for high-grade TB, while moderate tumor differentiation (OR = 0.341, 95% CI 0.129‒0.900, *p* =  0.030) and tumor stroma ratio ≤50% (OR = 0.228, 95% CI 0.081‒0.639, *p* =  0.005) were protective factors for high-grade TB. The relationships of TB to the various clinicopathological parameters are shown in Table 1 and Fig. 3.

**Table 1:** Tumor budding and clinicopathological parameters of 268 patients with LSCC.

*n* = 268		Chi-square tests analysis			Logistic regression	
	*n* (%)	LG-TB, *n* (%)	HG-TB, *n* (%)	*x*^2^/*p*	OR (95% CI)	*p*
Age median 62 (35–86 years), Mean ± SD (61.96 ± 9.117)				0.060/0.807		
<62	142 (53.0)	42/29.6	100/70.4			
≥62	126 (47.0)	39/31.0	87/69.0			
Gender				**5.798/0.016**		
Male	240 (89.6)	67/27.9	173/72.1		Reference	
Female	28 (10.4)	14/50.0	14/50.0		0.538 (0.175–1.659)	0.281
Histologic grade				**40.299/<0.001**		
Well-differentiated	64 (23.9)	34/53.1	30/46.9		Reference	
Moderately differentiated	142 (53.0)	46/32.4	96/67.6		0.341 (0.129‒0.900)	**0.030**
Poorly differentiated	62 (23.1)	1/1.6	61/98.4		1.223 (0.209–3.665)	0.508
Tumor cell nest size				**105.111/<0.001**		
Large >15	59 (22.0)	49/83.1	10/16.9		Reference	
Intermediate 5–15	162 (60.5)	31/19.1	131/80.9		2.644 (2.071–4.029)	**<0.001**
Small <5	47 (17.5)	1/2.1	46/97.9		2.829 (1.119–5.135)	**0.001**
Mitotic figure				**7.345/0.007**		
>15/10 HPFs	162 (60.4)	39/21.7	123/78.3		Reference	
1‒15/10 HPFs	106 (39.6)	42/36.6	64/63.4		0.522 (0.212–1.286)	0.157
Tumor stroma ratio				**6.876/0.009**		
>50%	115 (42.9)	25/21.7	90/78.3		Reference	
≤50%	153 (57.1)	56/36.6	97/63.4		0.228 (0.081‒0.639)	**0.005**
PT stage				**49.643/<0.001**		
PT1	77 (28.7)	44/57.1	33/42.9		Reference	
PT2	82 (30.6)	27/32.9	55/67.1		1.003 (0.366–2.747)	0.995
PT3+4	109 (40.7)	10/9.2	99/90.8		1.571 (1.059–2.040)	**0.040**
PN stage				**7.591/0.006**		
PN0	214 (79.9)	72/34.3	138/65.7		Reference	
PN1+N2	54 (20.1)	9/15.5	49/84.5		0.572 (0.132–2.479)	0.455
Angiolymphatic invasion				**3.997/0.046**		
No	219 (81.7)	72/32.9	147/67.1		Reference	
Yes	49 (18.3)	9/18.4	40/81.6		0.325 (0.071–1.484)	0.147
Perineural invasion				0.859/0.354		
No	245 (91.4)	76/31.0	169/69.0			
Yes	23 (8.6)	5/21.7	18/78.3			
Stromal TILs				**104.461/<0.001**		
>50%	51 (19.0)	44/86.3	7/13.7		Reference	
11%‒50%	137 (51.1)	34/24.8	103/75.2		2.785 (1.914–3.960)	**0.001**
0%‒10%	80 (29.9)	3/3.8	77/96.2		2.120 (1.512–3.980)	**<0.001**

LSCC, Laryngeal Squamous Cell Carcinoma; HG-TB, High-Grade Tumor Budding; LG-TB, Low-Grade Tumor Budding; OR, Odds Ratio; 95% CI, Confidence Interval 95%; SD, Standard Deviation; PT, Pathological-T; PN, Pathological-N; TILs, Tumor Infiltrating Lymphocytes.

Note: Bold fonts in Tables indicated the results were statistically significant.

### Survival analysis

The average follow-up period was 48.77 ± 26.44 months (range 10–192 months), and the median period was 44 months. During follow-up, 76 (28.4%) patients had tumor recurrence and 80 (29.8%) patients died.

Among the patients with no TB, 1–6 TB, and more than 6 TB (high-grade TB), univariate Cox survival analysis indicated that there was no statistical difference in DFS and OS between the patients with no TB and those with 1–6 TB (*p* >  0.05); there were statistically significant differences in DFS and OS between the more than 6 TB (high-grade TB) and the no TB, and between the more than 6 TB (high-grade TB) and the 1–6 TB (*p* <  0.05) (Supplementary Table and Supplementary Fig. 5A‒B).

**Figure 4:**
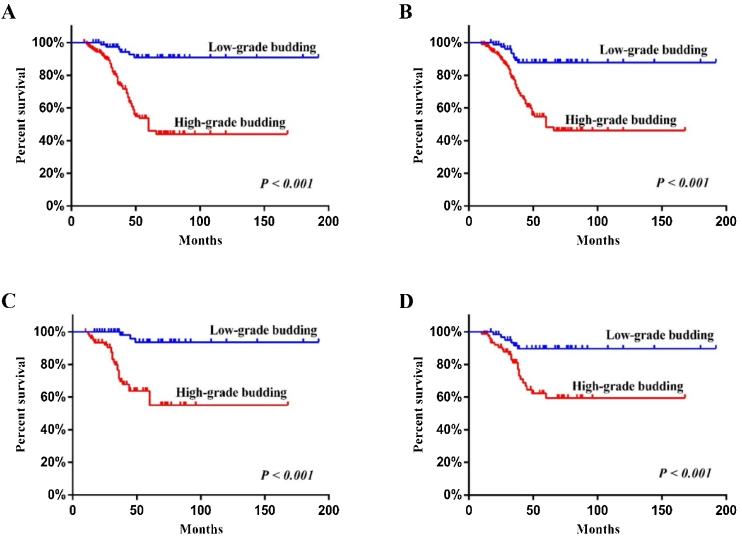
The effect of tumor budding on the survival of patients with LSCC illustrated by Kaplan-Meier curves: (A) disease-free survival; (B) overall survival; (C) disease-free survival in patients with T1-2N0 LSCC; (D) overall survival in patients with T1-2N0 LSCC.

In the low-grade TB and high-grade TB, univariate Cox survival analysis indicated that poorly differentiated tumors, small and intermediate tumor cell nest, high mitotic count, tumor stroma ratio ≥50%, higher pT stage, stromal TILs 0%‒10%, and high-grade TB were associated with poor DFS (*p* <  0.05) (Table 2 and Fig. 4A). Multivariate Cox survival analysis revealed that high-grade TB (HR = 7.566, 95% CI 2.529–22.638, *p* <  0.001) was independent risk factors for DFS (Table 2).

**Table 2:** Univariate and multivariate survival analysis of DFS in 268 patients with LSCC.

Prognostic factors	Univariate analysis		Multivariate analysis	
	HR (95% CI)	*p*	HR (95% CI)	*p*
Age ≥ 62 years vs. <62 years	0.934 (0.594–1.469)	0.769		
Female vs. male	1.010 (0.485–2.101)	0.980		
Histologic grade				
Moderately vs. well	1.430 (0.774–2.640)	0.253	0.638 (0.275–1.481)	0.296
Poorly vs. well	2.513 (1.294–4.880)	**0.007**	0.619 (0.232–1.648)	0.337
Tumor cell nest size				
Small vs. large	4.789 (2.115–10.842)	**<0.001**	1.649 (0.549–4.960)	0.373
Intermediate vs. large	3.024 (1.425–6.417)	**0.004**	1.221 (0.444–3.359)	0.699
Mitotic figure ≥15% vs. <15%	1.850 (1.110–3.084)	**0.018**	1.231 (0.629–2.408)	0.545
Tumor stroma ratio ≥50% vs. <50%	1.704 (1.084–2.678)	**0.021**	0.857 (0.465–1.577)	0.619
PT stage				
PT2 vs. pT1	1.585 (0.788–3.188)	0.196	1.058 (0.443–2.525)	0.899
PT3 vs. pT1	3.046 (1.559–5.952)	**0.001**	1.528 (0.606–3.855)	0.369
PT4 vs. pT1	4.004 (1.935–8.281)	**<0.001**	1.668 (0.590–4.717)	0.335
PN1+N2 vs. pN0	1.338 (0.803–2.230)	0.263		
Angiolymphatic invasion (Yes vs. No)	1.485 (0.875–2.522)	0.143		
Perineural invasion (Yes vs. No)	1.655 (0.851–3.220)	0.138		
Stromal TILs				
0%‒10% vs. >50%	4.337 (2.023–9.298)	**<0.001**	0.866 (0.264–2.842)	0.813
11%‒50% vs. >50%	1.549 (0.705–3.401)	0.276	0.410 (0.133–1.260)	0.120
HG-TB vs. LG-TB	7.815 (3.383–18.050)	**<0.001**	7.566 (2.529–22.638)	**<0.001**

LSCC, Laryngeal Squamous Cell Carcinoma; DFS, Disease-Free Survival; HG-TB, High-Grade Tumor Budding; LG-TB, Low-Grade Tumor Budding; HR, Hazard Ratio, 95% CI, Confidence Interval 95%; PT, Pathological T; PN, Pathological N; TILs, Tumor Infiltrating lymphocytes.

Note: Bold fonts in Tables indicated the results were statistically significant.

In addition, univariate Cox survival analysis revealed that moderately and poorly differentiated tumors, small and intermediate tumor cell nest, higher pT stage, angiolymphatic invasion, stromal TILs 0%‒50%, and high-grade TB were associated with poor OS (*p* <  0.05) (Table 3 and Fig. 4B). Multivariate Cox survival analysis showed that high-grade TB (HR = 4.279, 95% CI 1.474–12.422, *p* =  0.008), moderate tumor differentiation (HR = 2.766, 95% CI 1.087–7.040, *p* =  0.033), and poor tumor differentiation (HR = 3.909, 95% CI 1.381–11.061, *p* =  0.010) were found to be independent risk factors for OS (Table 3).

**Table 3:** Univariate and multivariate survival analysis of OS in 268 patients with LSCC.

Prognostic factors	Univariate analysis		Multivariate analysis	
	HR (95% CI)	*p*	HR (95% CI)	*p*
Age ≥ 62 years vs. <62 years	1.036 (0.668–1.606)	0.876		
Female vs. male	0.696 (0.303–1.599)	0.393		
Histologic grade				
Moderately vs. well	3.115 (1.399–6.937)	**0.005**	2.766 (1.087–7.040)	**0.033**
Poorly vs. well	6.092 (2.675–13.876)	**<0.001**	3.909 (1.381–11.061)	**0.010**
Tumor cell nest size				
Small vs. large	3.675 (1.582–8.537)	**0.002**	1.120 (0.384–3.269)	0.836
Intermediate vs. large	3.377 (1.605–7.108)	**0.001**	1.814 (0.731–4.498)	0.199
Mitotic figure ≥15% vs. <15%	0.993 (0.635–1.554)	0.976		
Tumor stroma ratio ≥ 50% vs. < 50%	1.116 (0.718–1.735)	0.624		
PT stage				
PT2 vs. pT1	1.689 (0.825–3.455)	0.152	0.854 (0.368–1.983)	0.714
PT3 vs. pT1	3.257 (1.645–6.452)	**0.001**	1.295 (0.557–3.014)	0.548
PT4 vs. pT1	5.069 (2.484–10.345)	**<0.001**	1.316 (0.509–3.402)	0.571
PN1+N2 vs. pN0	1.597 (0.990–2.576)	0.055		
Angiolymphatic invasion (Yes vs. No)	1.768 (1.082–2.889)	**0.023**	1.250 (0.716–2.184)	0.433
Perineural invasion (Yes vs. No)	1.201 (0.578–2.495)	0.623		
Stromal TILs				
0%‒50% vs. >50%	2.233 (1.115–4.474)	**0.023**	0.423 (0.148–1.211)	0.109
HG-TB vs. LG-TB	4.988 (2.484–10.015)	**<0.001**	4.279 (1.474–12.422)	**0.008**

LSCC, Laryngeal Squamous Cell Carcinoma; OS, Overall Survival; HG-TB, High-Grade Tumor Budding; LG-TB, Low-Grade Tumor Budding; HR, Hazard Ratio; 95% CI, Confidence Interval; PT, Pathological T; PN, Pathological N; TILs, Tumor Infiltrating Lymphocytes.

Note: Bold fonts in Tables indicated the results were statistically significant.

### Survival analysis of T1-2N0 LSCC

In 141 cases of T1-2N0 LSCC, 63 (44.7%) had low-grade budding, and 78 (55.3%) had high-grade budding. Multivariate Cox survival analysis showed that high-grade TB (HR = 13.052, 95% CI 1.161–146.732, *p* =  0.037) (Fig. 4C) and small tumor cell nest (HR = 11.962, 95% CI 1.083–132.156, *p* =  0.043) were independent prognostic factors for DFS in T1-2N0 LSCC (Table 4). In addition, high-grade TB (HR = 7.202, 95% CI 1.637–31.685, *p* =  0.009) (Fig. 4D), moderate tumor differentiation (HR = 2.844, 95% CI 1.074–7.534, *p* =  0.035), and angiolymphatic invasion (HR = 6.339, 95% CI 1.886–21.301, *p* =  0.003) were found to be independent prognostic factors for OS in T1-2N0 LSCC (Table 4).

**Table 4:** Multivariate survival analysis of DFS and OS in patients with T1-2N0 LSCC.

Prognostic factors	DFS		OS	
	HR (95% CI)	*p*	HR (95% CI)	*p*
Histologic grade				
Moderately vs. well	0.748 (0.278–2.015)	0.566	2.844 (1.074–7.534)	**0.035**
Poorly vs. well	0.977 (0.196–4.878)	0.977	4.425 (0.880–22.240)	0.071
Tumor cell nest size				
Small vs. large	11.962 (1.083–132.156)	**0.043**	1.094 (0.240–4.989)	0.908
Intermediate vs. large	5.952 (0.630–56.204)	0.119	1.328 (0.411–4.285)	0.635
Mitotic figure (≥15% vs. <15%)	1.014 (0.381–2.701)	0.978	0.914 (0.409–2.041)	0.826
Stromal TILs				
0‒10% vs. > 50%	0.413 (0.025–6.776)	0.535	0.168 (0.024–1.197)	0.075
11%‒50% vs. > 50%	0.273 (0.021–3.498)	0.319	0.285 (0.059–1.381)	0.119
Angiolymphatic invasion (Yes vs. No)	0.919 (0.118–7.159)	0.936	6.339 (1.886–21.301)	**0.003**
HG-TB vs. LG-TB	13.052 (1.161–146.732)	**0.037**	7.202 (1.637–31.685)	**0.009**

LSCC, Laryngeal Squamous Cell Carcinoma; DFS, Disease-Free Survival; OS, Overall Survival; HG-TB, High-Grade Tumor Budding; LG-TB, Low-Grade Tumor Budding; HR, Hazard Ratio; 95% CI, Confidence Interval 95%; TILs, Tumor Infiltrating Lymphocytes.

Note: Bold fonts in Tables indicated the results were statistically significant.

## Discussion

The earliest and greatest volume of research on TB has been carried out for colorectal adenocarcinoma. Other solid tumors, including those of the lung cancer,^9^ head and neck cancer,^6^, ^14^ and breast cancer,^11^ have also been studied. The frequency of TB in solid tumors is variable. Oh^19^ found that among 4196 cases of colon cancer, there were 1927 cases of TB, accounting for 45.9%, and Qi^20^ found that TB occurred in 142 of 153 cases of intestinal-type gastric cancer, accounting for about 92.8%. Xie^21^ found that the incidence of TB in oral tongue squamous cell carcinoma was 86.28%, and similarly Ekmekci^22^ found 62 out of 81 cases of LSCC had TB, accounting for about 76.5%. Our study included the largest number of LSCC cases so far, and we found that 226 out of 268 LSCC cases had TB. With a frequency of 84.3%, our results are the closest to those of Xie in the literature.

Currently, the assessment of TB is based on the method of the 2016 ITBCC recommendation.^18^ The number of TB in the “hot spots” at the front of the tumor invasions were counted and classified as, 0–4 for low-grade TB, 5–9 for intermediate TB, and ten or more than 10 for high-grade TB. For LSCC specifically, some authors have classified the proportion of TB to the entire tumor invasive edge as follows: less than 1/3 as mild budding, 1/3 to 2/3 as moderate budding, and more than 2/3 as severe budding.^22^, ^23^, ^24^ Zhang^15^ counted the total number of TB in 10 HPFs and the maximum budding in each field of 10 HPFs, and 10 buddings were the cutoff value for the TB groupings. Some studies have even selected 14 as the TB cutoff value.^14^, ^25^

In our study, ROC curve analysis calculated the optimal TB cutoff value for predicting patients' recurrence and death, and thus TB was divided into low-grade (0–6 TB/0.785 mm^2^) or high-grade (≥7 TB/0.785 mm^2^) according to this result. At present, there is no standardized method for TB evaluation in LSCC, however, although the TB grading methods used in ours and the aforementioned LSCC studies are all based on the ITBCC. We look forward to multi-center collaboration and larger sample studies to develop uniform and standardized methods for TB counting in LSCC.

Previous studies have found that TB is a histomorphologic characteristic related to the invasion and metastasis of colorectal adenocarcinoma. Few studies have shown that TB may be associated with the clinicopathological parameters of LSCC.^14^, ^24^, ^25^, ^26^ Our study used logistic regression to analyze the risk factors related to TB. Our results found that smaller tumor cell nest was the risk factors for high-grade TB, which was consistent with the results of Boxberg,^14^ who believed that tumor cell nest is the maximum degree of dissociation of tumor tissue into the stroma, and TB may be considered as the ability of tumor tissue to separate. We thus could not agree more with Boxberg. Smaller cell nests scattered in the tumor stroma have extensive spatial contact with the tumor stroma and may be more susceptible to the influence of tumor microenvironment, leading to epithelial mesenchymal transformation and enhanced tumor invasion.^27^

TILs are an important component of tumor microenvironment, and the high levels of stromal TILs have been shown to predict a better outcome than the poor stromal TILs in triple-negative and human epidermal growth factor receptor 2-overexpressing breast cancer.^17^ The relationship between TB and TILs is less studied in LSCC, but it is a very interesting topic as it may represent the interaction between tumor cells and host immune cells in promoting and inhibiting tumor growth.^28^ The studies of Zhang^29^ revealed that there was a negative correlation between the density of TB and TILs in the budding area of gastric adenocarcinoma, and there was a weak immune surveillance around the TB. Kadota^30^ found that TB in lung cancer was relevant to stromal TILs, especially FoxP3+ lymphocyte, which is a kind of regulatory T-lymphocytes, and may exert immunosuppression in tumor microenvironment. Our study showed that the low levels of stromal TILs were associated with high-grade TB in LSCC, which was consistent with the results of the above studies. Our results together with those reported in literature suggested that the number of TB and the density of TILs might be mutually exclusive relationship. In addition, some authors have also found that the combination of high-grade TB and low levels of TILs may predict the poorer prognosis of tumors.^31^, ^32^, ^33^ However, the immunophenotypes of TILs surrounding TB, as well as their effects on the prognosis of laryngeal cancer, are unclear and require further study.

Many studies have also shown that high-grade TB predicts poor survival in digestive tract adenocarcinoma.^7^, ^8^, ^10^, ^19^ A study of stage II colon cancer found that the 5-year DFS of the high-grade TB group was significantly lower than that of the low-grade TB group, and TB was an independent predictor of survival in stage II colorectal cancer.^34^ Stage II colorectal cancer patients with high-grade TB may also be considered for adjuvant therapy.^18^ However, there are few studies about the effects of TB on the survival of LSCC^15^, ^23^, ^24^, ^26^ or early laryngeal cancer patients. Our multivariate Cox survival analysis showed that patients with high-grade TB were an independent risk factor for poor DFS and OS in LSCC and T1-2N0 LSCC as well.

The significance of evaluating TB in patients with LSCC is that the survival time of patients with high-grade TB is short, and high-grade TB may be a potential important risk factor for tumor recurrence and death in LSCC. Therefore, it is necessary to include TB in the evaluation system of predictive survival factors for LSCC, and to supplement the existing prognostic evaluation system. In addition, a large number of studies have suggested that TB may be related to a variety of signal transduction pathways that promote tumor progression.^35^ TB cells may activate the TGFβ signal pathway and may be a promising drug target for the inhibition of oral cancer.^36^

Our study has several limitations, however. First, it is a retrospective study, and further prospective data are needed to bear out the prognostic value of TB as a morphological marker of LSCC. Second, due to the lack of detailed subtype information in the clinical diagnosis and pathological reports, we could not divide LSCC into supraglottic, glottic, and subglottic types. Finally, our study did not distinguish between HPV-associated SCC and HPV-independent SCC. Further study of TB in HPV-associated SCC and HPV-independent SCC is warranted but requires the accumulation of cases of HPV-associated SCC.

## Conclusion

In conclusion, we found that high-grade TB closely related to smaller tumor cell nest, higher pT stage, and the low levels of stromal TILs in LSCC. High-grade TB was an adverse risk factor that affects not only the DFS and OS of LSCC patients but also the survival of T1-2N0 LSCC patients. We thus recommend TB as a predicator of survival for laryngeal cancer and early laryngeal cancer.

## Authors’ contributions

Honggang Liu: Made substantial contributions to conception and design of the study; Revised the manuscript and gave final approval of the version to be published.

Li Luo: Involved in acquisition and analysis of data, and drafted the manuscript.

## Funding

This research did not receive any specific grant from funding agencies in the public, commercial, or not-for-profit sectors.

## Conflicts of interest

The authors declare no conflicts of interest.
