# Supplementary material for: High-grade tumor budding is a risk factor for survival in patients with laryngeal squamous cell carcinoma
Source: Braz J Otorhinolaryngol. 2023 Aug 22;89(5):101310. doi: 10.1016/j.bjorl.2023.101310 (PMC10495643; doi:10.1016/j.bjorl.2023.101310)
Supplement: Supplementary file 1 [file mmc1.doc]

BJORL-D-23-00122_Supplementary Material

**Supplementary Table** Univariate survival analysis of DFS and OS in three group of TB.

| **Prognostic factors** | **DFS** | | **OS** | |
| --- | --- | --- | --- | --- |
|  | **HR (95% CI)** | **p** | **HR (95% CI)** | **p** |
| 1‒6 TB vs. no TB | 0.620 (0.114‒3.386) | 0.581 | 1.022 (0.312‒3.348) | 0.972 |
| ≥ 7 TB (HG-TB) vs. no TB | 6.163 (2.244‒16.930) | **< 0.001** | 3.816 (1.652‒8.815) | **0.002** |

DFS, Disease Free Survival; OS; Overall Survival; HG-TB, High-Grade Tumor Budding; HR, Hazard Ratio; 95% CI, Confidence Interval 95%.

**Supplementary Figure 5** The effect of three group of tumors budding on the survival of patients with LSCC illustrated by Kaplan-Meier curves: (A) Disease-free survival; (B) Overall survival.
