# Supplementary figures and images for: High-grade tumor budding is a risk factor for survival in patients with laryngeal squamous cell carcinoma
Source: Braz J Otorhinolaryngol. 2023 Aug 22;89(5):101310. doi: 10.1016/j.bjorl.2023.101310 (PMC10495643; doi:10.1016/j.bjorl.2023.101310)

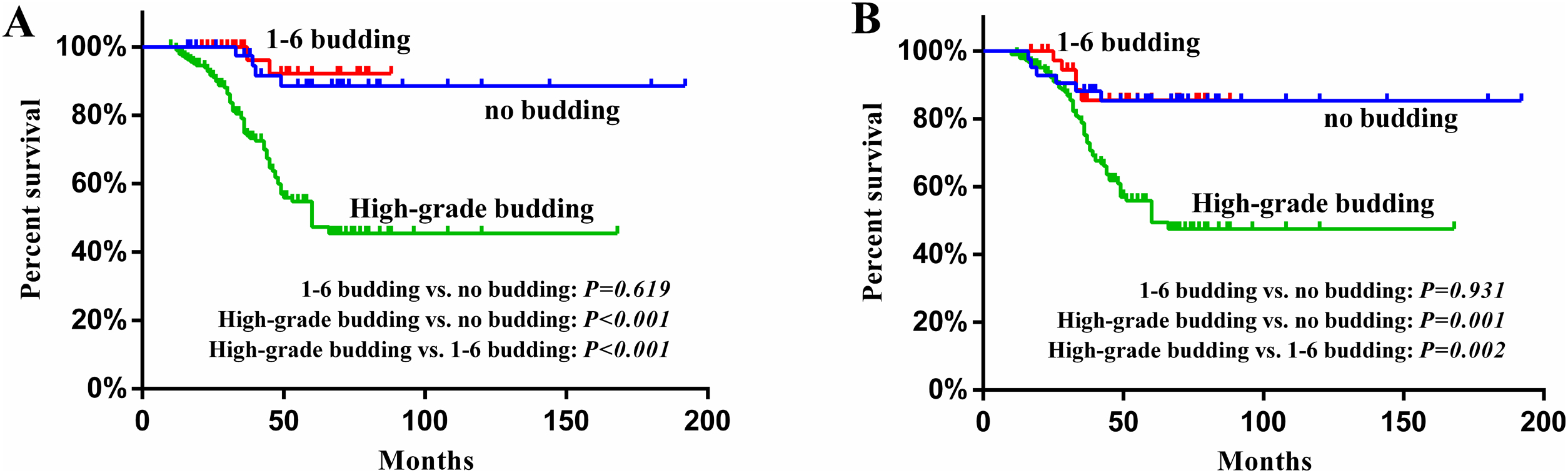

Supplement: Supplementary file 2 [file mmc2.jpg]
